# Epidemiology of multidrug-resistant tuberculosis (MDR-TB) in Ethiopia: a systematic review and meta-analysis of the prevalence, determinants and treatment outcome

**DOI:** 10.1186/s40794-018-0065-5

**Published:** 2018-06-14

**Authors:** Tadele Girum, Ebrahim Muktar, Kifle Lentiro, Habtamu Wondiye, Misgun Shewangizaw

**Affiliations:** 1 Department of Public health, college of Medicine and Health Sciences, Wolkite University, Wolkite City, Ethiopia; 2 Institute of Public health, college of Medicine and Health Sciences, Bahir Dar University, Bahir Dar City, Ethiopia; 3 Department of Public health, college of Medicine and Health Sciences, Arba Minch University, Arba Minch City, Ethiopia

**Keywords:** MDR-TB, Tuberculosis, Drug resistance, Treatment outcome

## Abstract

**Introduction:**

The emergence of MDR-TB remained a major public health threat particularly in developing countries. With increased prevalence and complexity of treatment, the burden of MDR-TB challenged the country. It is of an important; the epidemiology of drug resistant TB is not well understood. There are few studies conducted to assess the prevalence, determinants and treatment outcome of MDR-TB with inconclusive finding. Therefore, we aimed to conduct a systematic review and meta-analysis on Epidemiology of MDR-TB in Ethiopia, So that policy makers and other stalk holders could have pooled evidence on the problem to make a decision.

**Methods:**

The review was conducted through a systematic literature search of articles published between 1997 and 2017. Five bibliographic databases and libraries: PubMed/Medline, Global Health Database, Embase, the Cochrane Library, and African Index Medicus were used. After cleaning and sorting, analysis was performed using STATA version 11. The pooled rate of MDR-TB prevalence, determinants and treatment outcome was estimated with a random-effects model. Heterogeneity was assessed by the I^2^ and publication bias through funnel plot.

**Results:**

The 34 studies that were retained for final analysis enrolled a total of 7461 TB or MDR-TB patients. We found that 2.18% (95% CI 1.44–2.92%) of newly diagnosed and 21.07% (95% CI 11.47–30.67%) of previously treated patients have MDR-TB with overall prevalence of 7.24% (95% CI 6.11–8.37). History of previous treatment is the major determinant (pooled OR = 4.78 (95% CI 3.16–6.39)), while contact history and adherence also contributed. In this review the pooled death computed among 5 articles showed that 12.25% (95% CI 9.39–15.11%) of MDR-TB patients were died in the course of treatment. Complication, drug side effects and HIV infection were the main determinants for the death.

**Conclusion and recommendation:**

The prevalence is by far higher than the previous reports. It is mainly associated with history of previous treatment along with contact history. However, the treatment outcomes are comparable with previous studies, yet it is a concern. Comorbidities, drug side effects and HIV sero-positivity were the determinants. Thus, proper treatment of drug susceptible TB and early detection and treatment of MDR-TB before complication develops along with prevention of drug side effect and contacts with MDR-TB cases are very important.

## Background

Multidrug-resistant tuberculosis (MDR-TB) is a type of TB that is resistant to the two most effective first-line drugs; Rifampicin and Isoniazid. It results from primary infection or may develop in the course of a patient’s treatment [1]. The occurrence of MDR-TB is mainly attributable to human errors that predispose for resistance development, although genetic factors are believed to contribute to certain extent [2, 3]. The principal patient-related factor that predispose to MDR-TB is non-adherence to drug susceptible TB treatment [4].

Globally, 4.1% of new cases and 19% of previously treated TB cases were estimated to have had multidrug-resistant TB in 2016. In the same year, there were an estimated 600,000 incident cases of MDR/RR-TB with cases of MDR-TB accounting for 82% of the total and there were an estimated 350,000 MDR/RR-TB cases among notified TB patients. However only 153,119 cases were confirmed which is much lower than the estimated cases because only 41% of TB cases have been tested for resistance. There were about 240,000 deaths from MDR/RR-TB in 2016 [5].

In 2016, the 30 high MDR-TB burden countries accounted for 89.7% of the global incident cases of MDR-TB. While India, China and the Russian Federation alone accounted 47% of the global burden. In Africa, where little data is available, an estimated 93,000 cases were emerged in 2016 with estimated incidence of 2.7% among new and 14% among previously treated cases, but the vast majority of them went un-diagnosed. Ethiopia is one of the 30 MDR-TB high-burden countries, with an estimated 5800 cases reported in 2016 with the prevalence rate of 2.7% among new cases and 14% among previously treated patients [5, 6].

Globally, 129,689 of the 153,119 notified cases of multidrug-resistant TB and rifampicin resistant TB (MDR/RR-TB) in 2016 were enrolled in treatment. It means 85% of diagnosed cases or only 22% of the estimated incident MDR/RR-TB cases initiated treatment by the year. Which shows improvement in coverage and access to MDR TB treatment; however the cure rate is low and on the other hand mortality, default and failure are high. Only 50% of the MDR-TB patients in the 2012 cohort of detected cases were successfully treated, 16% died, and 10% failed [7]. Similarly, 54% of MDR/RR-TB cases in the 2014 cohort have successful outcome by 2016 [5].

The emergence of MDR-TB is a threat for the populations of resource-limited countries. Generally, the low socioeconomic status of the people, high prevalence of infectious diseases and limited access to well-equipped health care facilities worsens the effect of MDR-TB. Furthermore, poor treatment outcomes, longer treatment time (about two years), higher treatment costs, and many more complications make MDR-TB a more complex disease than TB [8, 9].

The overall epidemiology of drug resistant TB is not well understood in Ethiopia [10–12]. Accordingly there are few studies conducted to assess the prevalence, determinants and treatment outcome of MDR-TB with inconclusive findings. There are also other researches with finding of null impact of determinants on the prevalence and treatment outcome of MDR-TB. Therefore we aimed to conduct a systematic review on the epidemiology of MDR-TB in Ethiopia to assess the prevalence, determinants and treatment outcome of MDR-TB. So that policy makers and other stalk holders could have pooled evidence on the problem to make a decision.

## Methods

### Literature search strategy

This systematic reviews and meta analyses was conducted in accordance to the PRISMA (Preferred Reporting Items for Systematic Reviews and Meta-Analyses: guidance for reporting of systematic reviews and meta analyses) [13] through a systematic literature search of articles published between 1997 and 2017 containing information on MDR-TB prevalence, determinants and treatment outcome. Search engines, Electronic bibliographic databases and libraries: PubMed/Medline, Global Health Database, Embase, the Cochrane Library, African Index Medicus and Google scholar were used to retrieve articles published with in the study period. Combination of search terms: “Resistant TB”, “MDRTB”, “Isoniazid and rifampicin resistance”, “HIV and TB”, “Re-treatment and resistance”, “TB treatment” and the names of MDR-TB treatment hospitals were used with (AND, OR, NOT) Boolean (Search) Operators. In addition the reference lists of primary and pertinent review articles were also uploaded into an EndNote XI library (EndNote, Carlsbad, CA, USA) to identify cited studies not captured by the electronic search and after all checked for duplications.

### Selection /eligibility criteria

Studies that reported the prevalence of MDR-TB among new and/or previously treated patients, studies which compared two groups or reported determinants and studies which reported treatment outcome of MDR-TB that conducted in Ethiopia and published in English language regardless of the design and setting were used. Studies were also eligible for the review regardless of their study time. Studies conducted among presumptive MDR-TB cases only were excluded from the analysis to minimize over reporting. However studies conducted in MDR-TB referral centers which included both presumptive and all other cases were included.

### Operational definition

The following definition in accordance with the MDR-TB guide line [5, 14] were used in this meta-analysis

- Drug-resistant TB - TB that is resistant to any first-line anti-tuberculosis drug
- MDR-TB is TB that caused by strains of M.tuberculosis that are resistant to at least INH and RMP.
- MDR-TB among new cases (Primary drug resistance) is defined as resistance to isoniazid and rifampin drugs in patients that have never been treated for TB. This reflects person-to-person transmission of drug-resistant TB bacilli.
- MDRTB among previously treated TB patients (Acquired drug resistance) is defined as resistance to isoniazid and rifampin drugs in patients that have been treated for TB. This reflects drug resistance acquired during TB treatment but may also reflect infection or re- infection with resistant TB bacilli.
- Presumptive MDR-TB is as smear positive previously treated patients who define as relapse, return after default, and failure; new smear positive pulmonary TB patients whose sputum remains smear positive at month 2 or 3 of treatment; symptomatic close contacts of known MDR-TB patient, and new smear positive with Human Immunodeficiency Virus (HIV) infected patients.
- Medical complication: is a comorbid condition diagnosed as secondary diagnosis in tuberculosis patients

### Outcome of interest

This review was intended to measure the prevalence of MDR-TB among newly and/or previously treated TB patients. MDR-TB prevalence was calculated for the total sample and stratified for new and re-treatment groups. The odds of possible determinants were calculated for two variables and for others it was summarized. Lastly treatment outcome was measured in terms of mortality, cure and default rate.

### Data extraction and abstraction

The titles and abstracts derived through the primary electronic search were thoroughly assessed for possibility of reporting the intended outcome and filtered for potential eligibility. From each eligible research, the following information was extracted based on the preformed database (Excel, Microsoft, 2010) format: about author, characteristic of the study participants, studies (study design, cohort size, setting), prevalence of MDR-TB, Treatment outcome, year of publication, year of study start and end, eligibility criteria,, etc. All data were extracted independently and in duplicate using a standardized extraction form. Returned abstracts were reviewed and full texts retrieved if they contained relevant information. Mean way, each selected research was assessed for methodological quality and possibility of bias.

### Data analysis

After cleaning and sorting the final database was exported into Stata 11.0 for analysis (Stata, College Station, TX, USA). An outcome of interest was rate of MDR-TB among different groups, rate of mortality and their determinants. Estimate of MDR-TB prevalence was assessed for each study and standardized mean with 95% confidence interval was used. These were calculated with a random-effects model according to the DerSimonian and Laird method [15]. Heterogeneity was assessed by the I^2^ and values greater than 50% considered representing significant heterogeneity. When heterogeneity between studies was found to be significant, pooled estimates were based on random-effect models and the Hedges method of pooling. Results were displayed visually in forest plots. Bias was investigated by construction of funnel plots and Analysis was performed using the ‘metan’ and related functions in STATA version 11 (College Station, TX).

## Results

### Studies included

**Fig. 1:**
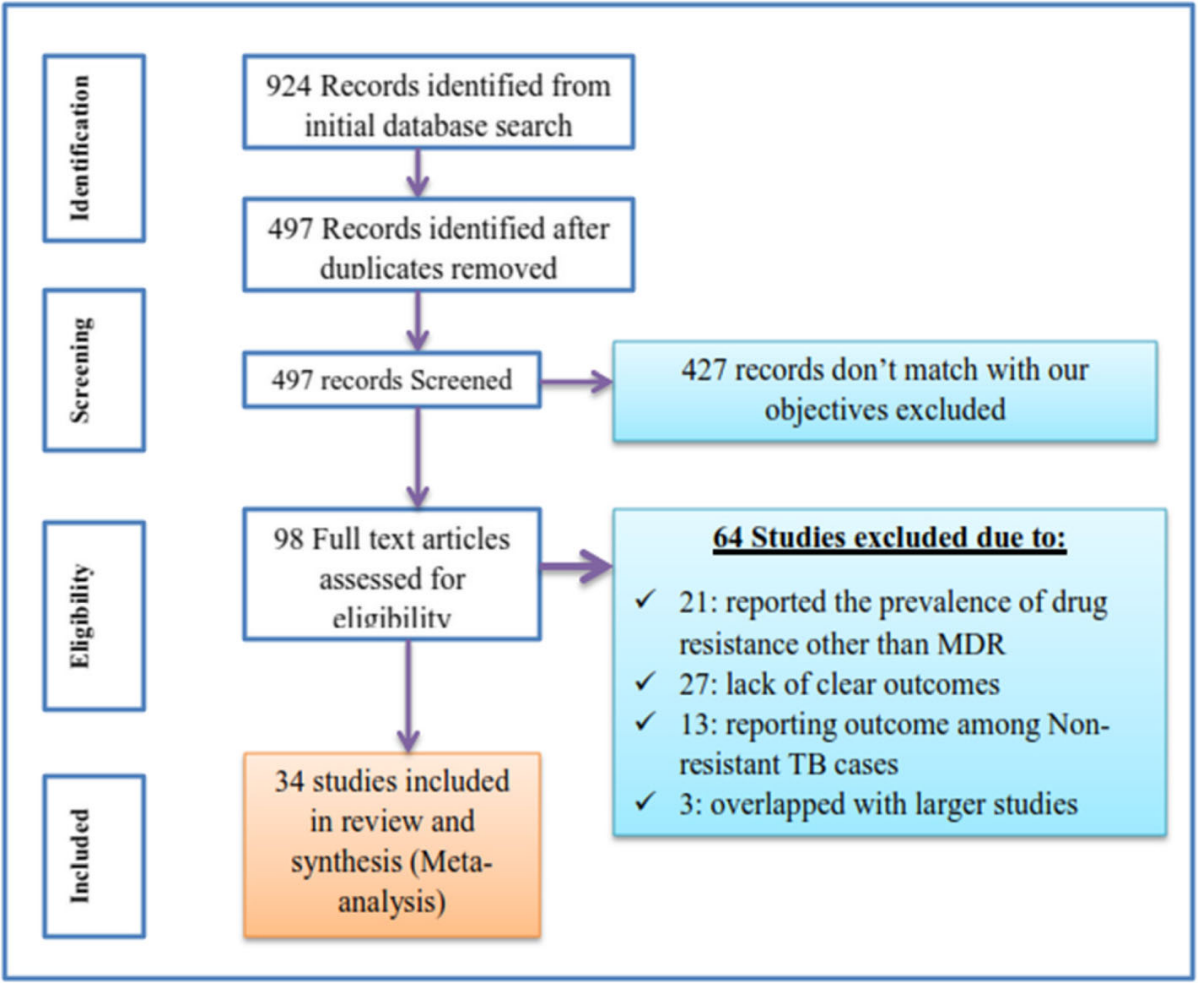
Flow chart for study search, selection and screening for the review

Our initial electronic database search with the chosen search terms identified 924 citations in the form of abstract, bibliography and full text research. Out of these, 427 researches which do not match with our objectives were excluded after reviewing their titles and abstracts. Then the remaining citations were transferred to endnote and cleaned for duplications and 98 articles were identified for full text review. Of the 98 articles reviewed in full text, 34 articles [16–49] were retained for final analysis based on the inclusion-exclusion criteria and quality assessment and 64 studies were removed prior to analysis for different reasons: 21 studies reported the prevalence of drug resistance other than MDR, 27 removed for lack of clear outcomes, 13 removed for reporting outcome among Non-resistant TB cases, 3 overlapped with larger studies. The study selection process is presented in (Fig. 1).

### Description of findings

The 34 studies that were retained for final analysis enrolled a total of 7461 TB or MDR-TB patients [16–49]. With duplicates (repeated count), 23 cross-sectional studies measured prevalence of MDR-TB among 3477 New and /or previously treated patients [16–38], 14 cross-sectional and case control studies assessed determinants of MDR-TB among 3534 patients [24, 26, 28, 32, 33, 35, 37–44] and the rest 5 cohort studies assessed the treatment outcome and its determinants among 1538 MDR-TB patients [45–49]. Except one, all the studies were institution based researches conducted in different regions of Ethiopia and published between 1997 and 2017 with English language. Thirteen studies were conducted in Addis Ababa, where two of the first MDR-TB treatment centers were located and the rest were conducted in other referral centers and TB treatment hospitals. Survey characteristics are described in (Table 1).

**Table 1:** Characteristics of studies included in the review

Studies included in the review	Study area	Study population	Study design	Study period	No of patients
[16] Demissie et al. 1997	Addis Ababa	New PTB patients	Cross-sectional study	1994	167
[17] Gebeyehu, 2001	Arsi Zone	PTB patients	Cross-sectional study	1997–98	195
[18] Bruchfeld, 2002	Addis Ababa	PTB patients	Cross-sectional study	2000	121
(19)Asmamaw et al., 2008	Addis Ababa	New PTB patients	Cross-sectional study	2004–5	173
[20] Meskel et al. 2008	Addis Ababa	Previously treated PTB	Cross-sectional study	2001–2	84
[21] Agonafir, 2010	Addis Ababa	PTB patients	Cross-sectional study	2005–6	107
[22] Abate et al., 2012	Addis Ababa	Previously treated PTB	Retrospective	2004–8	376
[23] Abebe et al. 2012	South western Ethiopia	New PTB patients	Cross-sectional study	2010–11	136
[24] Tessema et al. 2012	North Ethiopia	PTB patients	Cross-sectional study	2009	260
[25] Yimer et al. 2012	ARS major towns	New PTB patients	Cross-sectional study	2008	93
[26] Hussein et al. 2013	Bahirdar, Fitche & Ambo	PTB patients	Cross-sectional study	2011	102
[27] Biadglegne et al.2014	Bahirdar, Gondar & Dessie	TB lymphadenitis	Cross-sectional study	2012	226
[28] Esmael et al. 2014	Eastern ARS	PTB patients	Cross-sectional study	2010–11	230
[29] Daniel et al. 2014	Debre Birhan	PTB patients	Cross-sectional study	2013–14	40
[30] Seyoum et al. 2014	Eastern Ethiopia	New PTB patients	Cross-sectional study	2011–13	357
[31] Tekle et al. 2014	Benishangul & Awi	PTB patients	Cross-sectional study	2013–14	87
[32] Abdella et al., 2015	South western Ethiopia	Previously treated PTB	cross sectional	2012–13	79
[33] Adane et al. 2015	East Gojjam	PTB patients	Cross-sectional study	2011–12	89
[34] Maru et al. 2015	Dessie	PTB patients	Cross-sectional study	2012–13	118
(35)Mekonnen et al. 2015	North Gondar	PTB patients	Cross-sectional study	2014	124
[36] Ali et al. 2016	South Ethiopia	PTB patients	cross sectional	2013	109
[37] Hamussie et al. 2016	Arsi Zone	PTB patients	Cross-sectional study	2013–14	106
[38] Brhane et al., 2017	Jigjiga	PTB patients	cross sectional	NR	98
[39] Mulisa et al. 2015	Oromia Region	MDR & Non MDR TB	Case control study	2013–14	439
[40] Dessalegn et al. 2016	Addis Ababa	MDR & Non MDR TB	Case control study	NR	206
[41] Hirpa et al. 2013	Addis Ababa	MDR & Non MDR TB	Case control study	2011–12	268
[42] Deressa et al. 2014	Addis Ababa	MDR & Non MDR TB	Case control study	2013	384
[43] Assefa et al. 2017	Addis Ababa	MDR & Non MDR TB	Case control study	2013	710
[44] Tadesse et al. 2015	Addis Ababa	MDR & Non MDR TB	cross sectional	2008–11	439
[45] Molalign et al. 2015	Addis Ababa & Gondar	MDR TB	Cohort	2011–14	342
[46] Alene et al. 2016	North west Ethiopia	MDR TB	retrospective cohort	2010–15	242
[47] Getachew et al. 2013	Addis Ababa	MDR TB	retrospective cohort	2011–12	188
[48] Meressa et al. 2015	Addis Ababa	MDR TB	Cohort	2009–14	612
[49] Girum et al. 2017	South Ethiopia	MDR TB	retrospective cohort	2011–16	154

ARS- Amhara Regional state MDR- TB = Multidrug resistant tuberculosis

### Prevalence of MDR-TB

Of the 23 researches which reported MDR-TB prevalence, 14 researches reported prevalence of MDR-TB among previously treated patients, 14 researches reported among New PTB patients, 20 reported any first line drug resistance, 17 measured prevalence of isoniazid (INH) resistance and 16 reported prevalence of Rifampicin resistance (Table 2).

**Table 2:** Prevalence of primary and multidrug resistance

Studies included	Sample size			Primary drug resistance %			MDR % among		
	New	Re-treated	Total	Any drug	INH	RMP	New	re-treatment	over all
Demissie et al. 1997	167	0	167	15.6	8.4	1.8	NR	NR	1.2
Gebeyehu, 2001	195	19	195	19.5	7.6	0	NR	NR	0.5
Bruchfeld, 2002	103	18	121	14	8.3	2.5	NR	NR	0.8
Asmamaw et al., 2008	173	0	173	21.4	13.3	1.2	NR	NR	0.6
Meskel et al. 2008	0	84	84	53.6	NR	NR	NR	26.2	26.2
Agonafir, 2010	44	63	107	60.8	54.2	43.9	2.3	71.4	43
Abate et al., 2012	0	376	376	72.9	56.1	46.5		46.3	46.3
Abebe et al. 2012	136	0	136	18.4	13.2	2.2	1.5	NR	1.5
Tessema et al. 2012	214	46	260	15.8	13.8	5.8	3.7	10.9	5
Yimer et al. 2012	93	0	93	30.1	20.4	3.2	1.1	NR	1.1
Hussein et al. 2013	93	9	102	36.3	29.4	13.7	11.8	11.1	11.8
Biadglegne et al.2014	213	13	226	NR	NR	NR	1.4	0	1.3
Esmael et al. 2014	165	65	230	33.5	NR	NR	1.8	18.5	6.5
Daniel et al. 2014	NR	NR	40	NR	NR	NR	6.3	12.5	7.5
Seyoum et al. 2014	357	0	357	23	14	2.8	1.1	NR	1.1
Tekle et al. 2014	75	12	87	16.5	12.6	2.3	1.3	8.3	2.3
Abdella et al., 2015	79	0	79	58.6	51.4	32.9	NR	31.4	31.4
Adane et al. 2015	77	12	89	20.23	6.74	5.62	1.3	16.7	3.4
Maru et al. 2015	103	15	118	17.8	13.55	0	0	13.3	1.7
Mekonnen et al. 2015	88	36	124	NR	NR	NR	2.3	13.9	5.6
Ali et al. 2016	NR	NR	109	24.77	7.34	5.5	NR	NR	3.67
Hamussie et al. 2016	85	21	106	21.7	13.2	7.5	2.4	14.3	4.7
Brhane et al., 2017	67	31	98	18.4	NR	8.2	4.5	22.6	10.2
Weighted mean	–	–	–	31.1	21.65	13.4	2.18*	21.07*	7.24*
Total N	2527	820	3477	3087	2675	2460	2527	820	3477

INH-Isoniazid, RMP-rifampicin, NR = Not reported * value indicates polled prevalence from random effect model

**Fig. 2:**
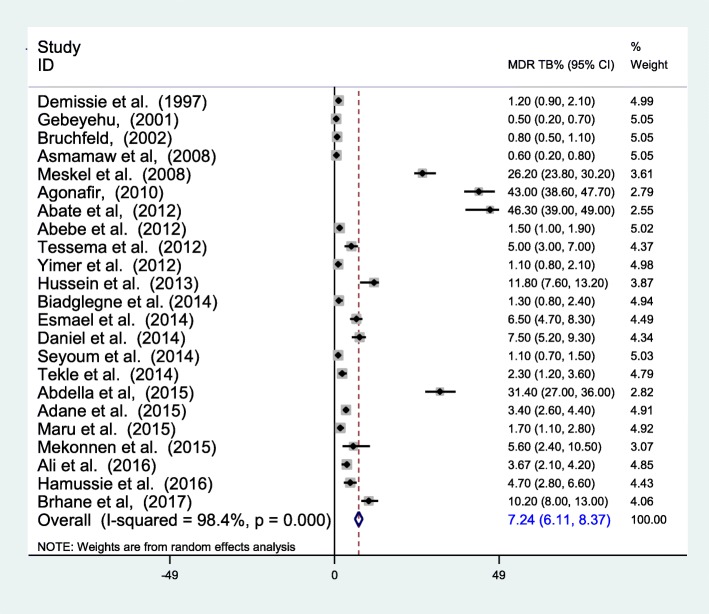
Forest plot showing the prevalence of MDR-TB among the total samples

Overall, the prevalence of MDR-TB ranged from 0.5 to 46.3%, with a pooled prevalence of 7.24% (95% CI 6.11–8.37) (The pooled estimate was from random effect analysis). The proportion of MDR-TB among all TB cases varies from place to place. Among the reviewed researches, six articles reported high prevalence of MDR-TB in the range of 10.2–46.3% [20–22, 26, 32, 38]; Three of them being reported in Addis Ababa [20–22]. The highest MDR-TB prevalence with a rate of 46.3% was reported by Abate et al. [22] in Addis Ababa among previously treated PTB patients and as high as a prevalence of 43% was also reported among New and Previously treated PTB patients by Agonafir et al. [21] (Fig. 2).

Studies conducted in other parts of the country also reported similar results. A study conducted in south western Ethiopia indicated that 31.4% of previously treated PTB patients have MDR-TB [32]. Additionally, 11.8% of MDR-TB was reported by Hussein et al. in 2013 [26] and 10.2% by Brhane et al. in 2017 [38] among all cases. On the other hand, three of these studies reported MDR-TB prevalence of below 1% [17–19]. .

**Fig. 3:**
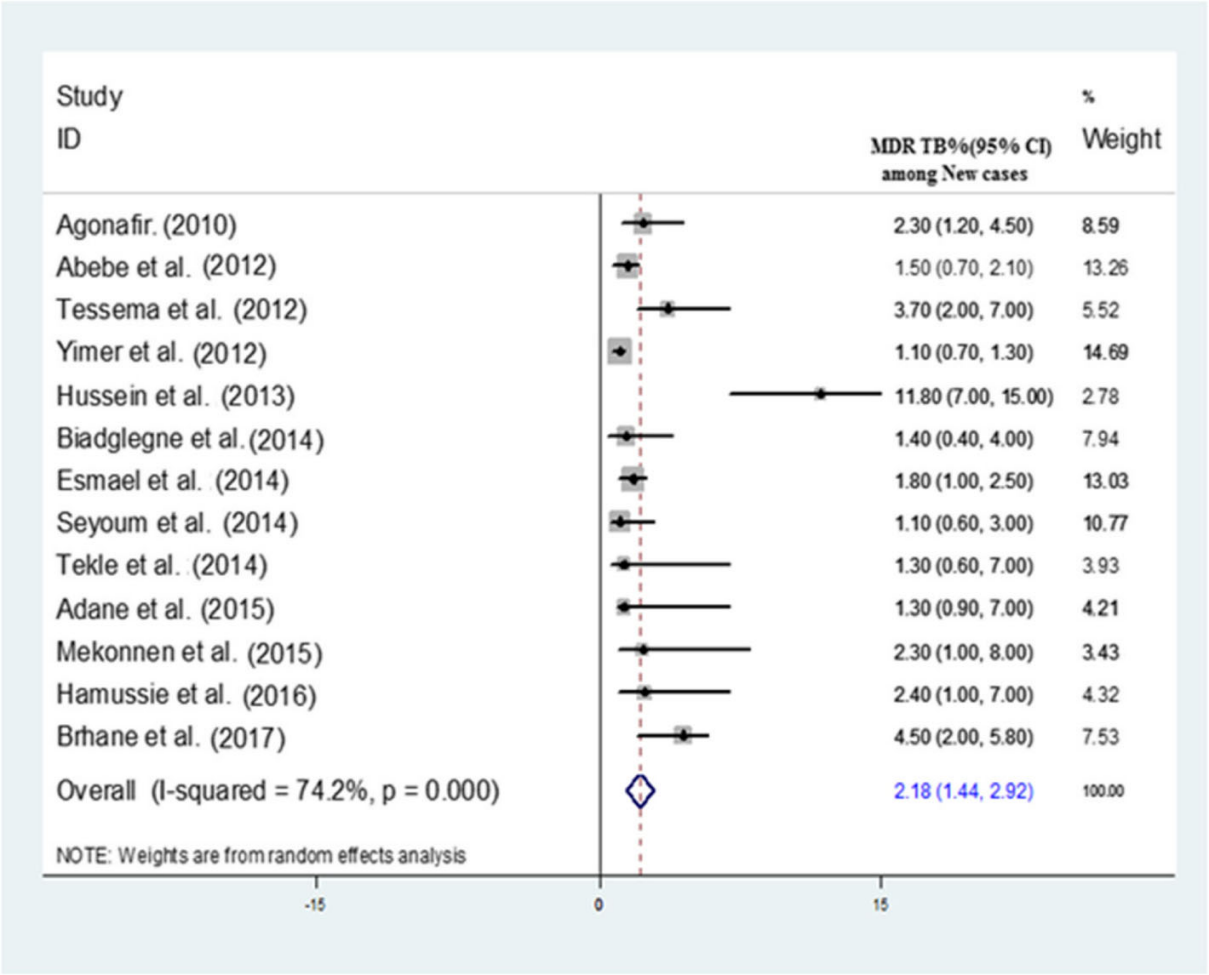
Forest plot showing the prevalence of MDR-TB among newly diagnosed TB cases

**Fig. 4:**
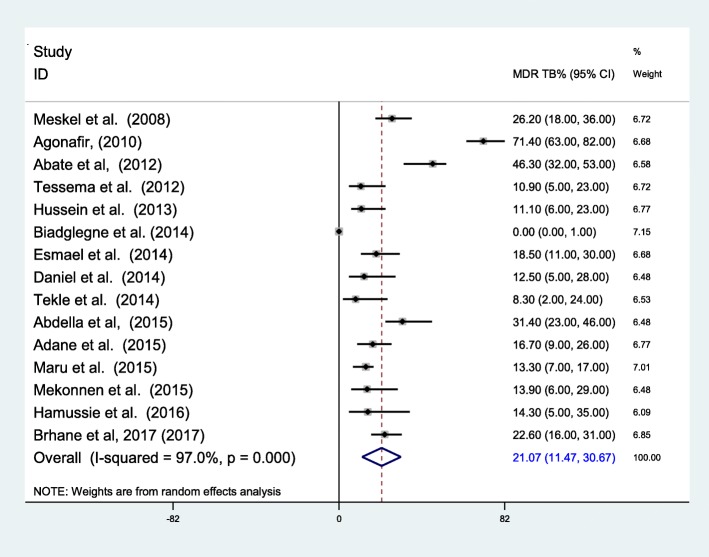
Forest plot showing the prevalence of MDR-TB among previously treated cases

The pooled prevalence of MDR-TB among newly diagnosed TB cases was 2.18% (95% CI 1.44–2.92%) (Fig. 3). The highest prevalence among new cases was reported to be 11.8 by Hussien et al. [26]. The overall prevalence of MDR-TB among previously treated TB cases was 21.07% (95% CI 11.47–30.67%) (Fig. 4). The heterogeneity test indicated that there is statistical significant variation among pooled estimate of MDR-TB in previously treated cases and new cases. Therefore, the pooled estimate was from random effect analysis in both cases. The observed variation of MDR-TB prevalence among newly diagnosed and previously treated cases was statistically significant (*P* < 0.001).

In our review, 20 researches that enrolled a total of 3087 patients assessed the prevalence of any first line anti TB drug resistance and 58.6% are found to be resistant. Of the 17 researches that assessed the prevalence of INH resistance, 51.4% were found to have resistant TB to INH. Similarly, 32.9% of the 2460 patients have Rifampicin resistance (Table 2).

### Determinants of MDR-TB

**Fig. 5:**
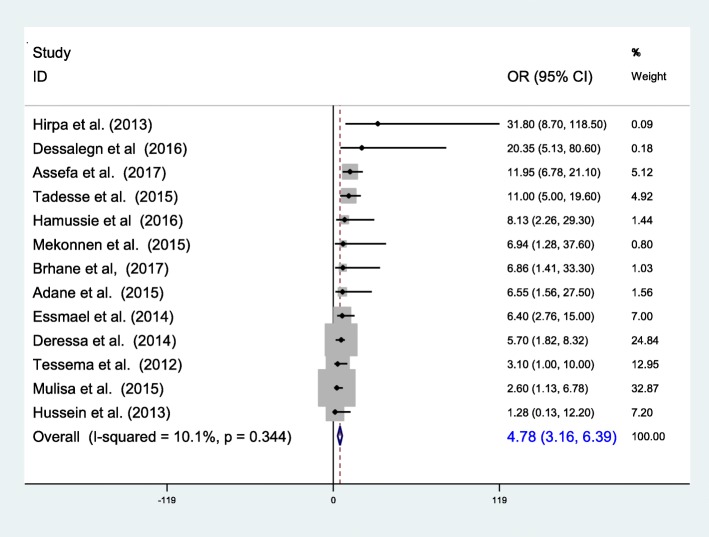
The pooled odds of previous treatment on MDR-TB prevalence

The reviewed articles indicated that previous exposure to TB treatment, contact history to known MDR-TB cases and previous treatment outcome were known be associated with prevalence of MDR-TB [24, 26, 28, 32, 33, 35, 37–44]. In this review, MDR-TB was found to be significantly associated with a previous history of anti-TB drug treatment. The risk of having MDR-TB was about 5 times higher in individuals with a previous history of anti-TB treatment. Except Hussien et al.’s report [26], all of the 12 researches reported that previous treatment is positively associated with MDR-TB with a pooled odds of 4.78 (95% CI 3.16–6.39) (Fig. 5).

**Fig. 6:**
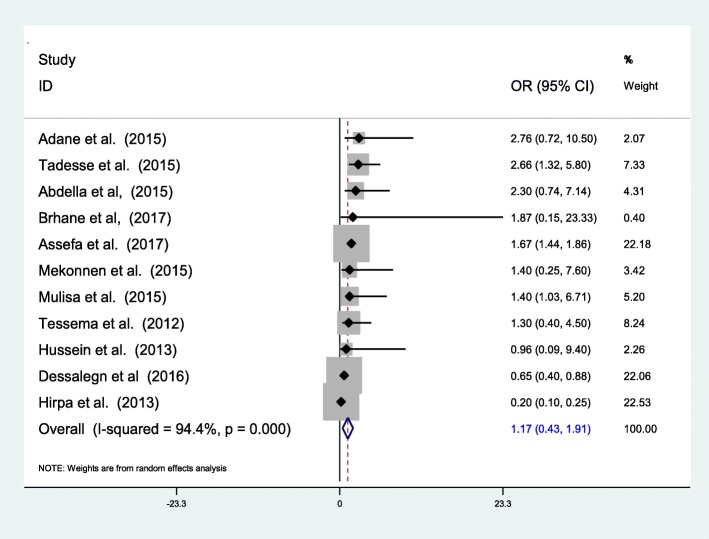
The pooled odds of HIV infection on MDR-TB prevalence

However, in this review HIV was not significantly associated with MDR-TB prevalence. Of the 11 studies which reviewed to evaluate the association between MDR-TB and HIV only 3 researches by Tadesse et al. [44], Mulisa et al. [39] and Assefa et al. [43] reported significant positive association. In contrast to these, another 3 researches [26, 40, 41] showed preventive association and 5 researches [24, 32, 33, 35, 38] sowed non-significant positive association. Over all pooled odds of HIV was 1.17 (95% CI 0.43–1.91) which indicates a positive but insignificant association (Fig. 6).

In addition to these factors, exposure to a known MDR-TB case (Contact history) in Mulisa et al. [39], Assefa et al. [43] and Brhane et al. [38], treatment adherence and drug side effect in Hirpa et al. [41] previous treatment outcome in Meressa et al. [48] were found to have a positive association with MDR-TB in Ethiopia. However a pooled estimate was not computed due to the smaller number of studies which assessed each variable.

### Treatment outcome of MDR-TB

**Fig. 7:**
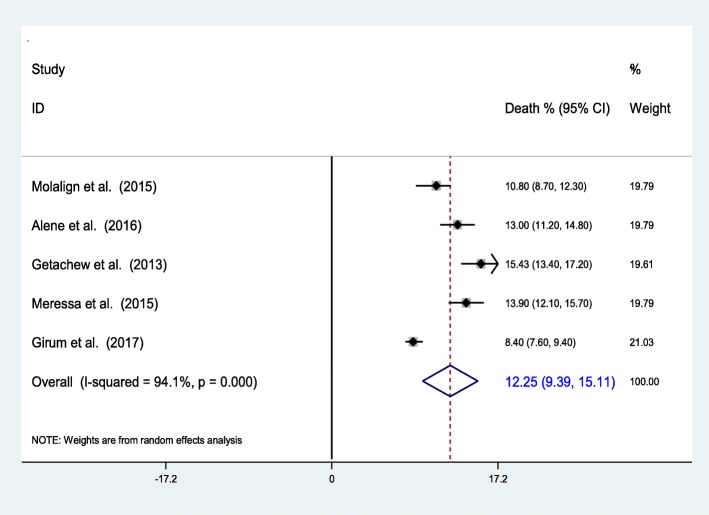
Forest plot showing the rate of mortality in the course of MDR-TB treatment

Concerning the outcomes, this meta-analysis revealed that 8.4–13.9% of MDR-TB patient died during treatment and 25–64.7% were get cured, another 10–16.9% were completed treatment [45–49]. According to Molalign et al. [45] of the 342 MDR-TB patients 10.8% were died and the median survival time was 16 months. In another study 13% of patients were died and 54% were cured [46]. A research by Girum et al. [49] also reported that 8.4% of patients were died, 25.3% were cured and 16.9% completed their treatment. In this review the pooled death computed among 5 researches showed that 12.25% (95% CI 9.39–15.11%) of MDR-TB patients were died (Fig. 7). Presence of medical complication, drug side effect, HIV sero-positivity and baseline weight were the most common reasons for the death [47, 49].

## Discussion

This study assessed the prevalence of MDR-TB among newly diagnosed and previously treated patients, identified possible determinants and evaluated treatment outcome by reviewing 34 researches conducted in Ethiopia. Within this review 23 researches were pooled together to measure prevalence of MDR-TB and 7.24% (95% CI 6.11–8.37) found to have MDR-TB with the rate of 2.18% (95% CI 1.44–2.92%) among new cases and 21.07% (95% CI 11.47–30.67%) among previously treated patients. History of previous treatment along with some other variables was the major determinants. During the course of treatment 12.25% (95% CI 9.39–15.11%) of MDR-TB patients were died mainly associated with complications.

According to this meta-analysis, the pooled prevalence of MDR-TB among newly diagnosed patients was comparable to the finding of previous meta-analysis by Eshetie et al. [50] where 2% (95% CI 1–2%) of newly diagnosed TB patients have MDR-TB. However, it is slightly higher than a previous meta-analysis report that was conducted in Sub-Saharan Africa countries where the prevalence among the new cases was 1.5% (95% CI 1.0–2.3) [51] and the national survey report of WHO estimate for Ethiopia [5, 52] and the report of national surveys in Benin [53]. On the other hand the prevalence of MDR-TB among new cases was lower than the first Ethiopian national TB survey report of 2.7% and the survey report of Mozambique and Rwanda [54–56].

The pooled prevalence of MDR-TB among previously treated patients was higher than both of the previously stated researches where 15% (95% CI 12 17%) of patients in Eshetie et al. [50] and 10.3% (95% CI 5.8–17.4%) of patients in Asres et al. [51] have MDR-TB. Likewise, anti-tuberculosis survey in Ethiopia, Benin, Mozambique and Rwanda showed that the prevalence of MDR-TB among previously treated patients was 17.9, 11.1, 11.2 and 9.4% [53–56] respectively which is by far lower than our pooled estimate.

This discrepancy may be related to the inclusion of many researches conducted in MDR-TB referral centers which knowingly or unknowingly included presumptive MDR-TB cases where the prevalence of MDR-TB is higher. It is logical that patients who have treatment history and currently on treatment are more likely to have MDR-TB than previously treated patient recruited in the community where their TB was treated. There for inclusion of larger samples in hospital based studies particularly in MDR-TB referral centers increases the finding of MDR-TB cases. However in newly diagnosed cases weather they are recruited in the community or in hospitals the prevalence is similar, that is why national surveys and institutional studies have comparable results. In addition to this difference in base line burden, difference in settings and quality of care may have explained the difference [50, 51].

In line to many of the previous reports [50, 51] MDR-TB was highly associated to history of previous TB treatment (OR = 4.78 (95% CI 3.16–6.39). The odds of developing MDR-TB among previously treated patients was 8.11 (7.52, 8.74) and 5.69 (3.61–8.96) in Ethiopia [50] and Sub-Saharan countries [51] respectively. As previously treated patients have the chance to acquire a new infection and develop resistance through gene mutation while up on treatment the likely hood of having MDR-TB compared to newly diagnosed TB patient is higher [5, 51].

The association between HIV and MDR-TB was not conclusive. Over all pooled odds of HIV was 1.17 (95% CI 0.43–1.91) which is a positive but insignificant association. Tadesse et al. [44], Mulisa et al. [39] and Assefa et al. [43] reported significantly positive association between HIV infection and MDR-TB. However, a significant positive association between HIV and MDRTB has not been reported from previous reviews in SSA [51] and study results in east Africa and Ethiopia [10, 22, 24, 38]. It is long been known that HIV suppress the human cellular immunity and shorten the period from TB infection to TB disease development [8–10]; however, its contribution to the development of drug resistant TB seems to be not significant.

According to the reports of Mulisa et al. [39], Assefa et al. [43] and Brhane et al. [38] exposure to a known MDR-TB case (Contact history) has a significant association with the prevalence of MDR-TB. It is also reported by Federal Ministry of Health of Ethiopia [57] that exposure to a known MDR-TB case along with history of using poor quality TB drugs and mal-absorption were found to have a positive association with MDR-TB in Ethiopia. Since one of the modes of transmission of MDR-TB is through droplet, a close contact history with a known MDR-TB patient carries higher risk for the development of the disease. Meanwhile, treatment adherence and drug side effect and previous treatment outcome were found to have a positive association with MDR-TB in Ethiopia [41, 48].

Overall, 65–80% of patients were completed, cured or alive on treatment throughout the period of follow up with in the reviewed researches [45–49] which met the WHO target of 75% [1] and it is higher than the achievement of most MDR-TB prevalent countries [5, 58]. Also multiples of recent systematic review and meta-analysis reported a lower success rate. Group based meta-analysis Reported pooled treatment success rate of 66.4% [59], also another individual patient based study review reported a success rate of lower than this fig. [60]. On the other hand some researches from Ethiopia and Haiti reported higher success rates [48, 61].

The proportion of death in our case is also comparable to many other studies [58–60]. However the treatment outcome from a cohort studies may differ depends on whether the research is an open cohort in which outcome may be experienced at the later phase of the treatment or closed cohort in which the outcome is experienced during or before the research is completed. In line to our report a result from a review of 35 articles and another individual patient review reported a death rate of greater than 10% [59, 60].

The treatment advancement through time, patient condition during admission, program organization, the setting and multitude of other factors may contribute significantly for difference in success rate. Generally in Ethiopia it was noted from WHO reports and previous studies that the success rate was higher [5]. Most researchers reported that poor outcome of MDR TB treatment is highly contributed by prevalence of HIV co-infection [45–49] and other medical complications. In addition to these, drug side effect and smaller baseline weight were significantly associated with mortality [49]. We have discovered the same relation in which the risk of death in HIV positive individuals was higher than sero-negatives. This is because during HIV infection the prevalence of co-infection, the probability of drug interaction and side effects and overall compliance will decrease which further increase mortality. Similarly, presence of complication contributes for mortality [47, 49].

The finding of this review may suffer from the fact that the research is from tuberculosis treatment centers their representativeness for the community is limited. The review was also limited to researches published in English and within the specified data sources as well as it is covering only a specific geographic areas, so that generalization for the country at large may be difficult. Due to institutional based nature of the primary researches inclusion of presumptive cases may be there, which could further increase the prevalent estimate.

## Conclusion and recommendation:

The review showed that MDR-TB is continued to become a serious public health problem in Ethiopia. The prevalence is by far higher than the previous reports among previously treated patients. Meanwhile history of previous treatment along with contact history contributed for the development of the disease. The treatment outcomes reported in this review was comparable with previous studies, yet it is a concern. The main predictors of mortality among MDR-TB patients up on treatment were presence of comorbidities, adverse side effects, mal-absorption, HIV sero-positivity and smaller baseline weight. Thus, proper treatment of drug susceptible TB and early detection and treatment of MDR-TB before complication developed along with prevention of drug side effect and contacts with MDR-TB cases are very important.

## Abbreviations

DST: Drug Susceptibility Testing
MDR: Multidrug Resistance
MDR-TB: Multidrug-Resistant Tuberculosis
PRISMA: Preferred Reporting Items for Systematic Reviews and Meta-Analyses
PTB: pulmonary Tuberculosis
RR: Rifampicin resistant
TB: Tuberculosis
